# *N*-Benzyl­pyridin-2-amine

**DOI:** 10.1107/S1600536810044478

**Published:** 2010-11-06

**Authors:** Jun Wang, Chuntao Dai, Jianhua Nie

**Affiliations:** a Zhongshan Polytechnic, Zhongshan, Guangdong 528404, People’s Republic of China

## Abstract

In the crystal of the title compound, C_12_H_12_N_2_, inter­molecular N—H⋯N hydrogen bonds form rings of graph-set motif *R*_2_^2^(8) and C—H⋯π inter­actions further consolidate the dimers. Neighbouring dimers are further connected into a three-dimensional network by C—H⋯π inter­actions. The benzyl and pyridyl rings form a dihedral angle of 67.2 (1)°

## Related literature

For general background to the topologies and potential applications of metal coordination polymers, see: Benelli & Gatteschi (2002 ▶). For related structures, see: Davies *et al.* (2001 ▶); Wan *et al.* (2004 ▶); Zhou & Richeson (1995 ▶). For bond-length data, see: Allen *et al.* (1987 ▶). For hydrogen-bonding graph-set motifs, see: Bernstein *et al.* (1995 ▶). For another report on the structure of *N*-benzyl­pyridin-2-amine, see: Wang & Zhao (2010 ▶).
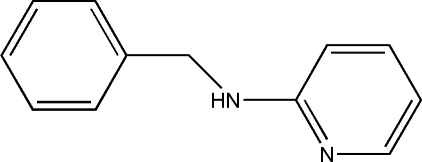


## Experimental

### 

#### Crystal data

C_12_H_12_N_2_
*M* *_r_* = 184.24
Triclinic, 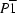

*a* = 5.9014 (16) Å
*b* = 8.025 (2) Å
*c* = 10.561 (3) Å
α = 95.471 (4)°
β = 91.244 (4)°
γ = 94.779 (3)°
*V* = 495.9 (2) Å^3^
*Z* = 2
Mo *K*α radiation
μ = 0.07 mm^−1^
*T* = 296 K
0.23 × 0.20 × 0.19 mm

#### Data collection

Bruker APEXII area-detector diffractometer
2551 measured reflections
1762 independent reflections
1387 reflections with *I* > 2σ(*I*)
*R*_int_ = 0.012

#### Refinement

*R*[*F* ^2^ > 2σ(*F* ^2^)] = 0.042
*wR*(*F* ^2^) = 0.115
*S* = 1.07
1762 reflections
127 parameters
H-atom parameters constrained
Δρ_max_ = 0.23 e Å^−3^
Δρ_min_ = −0.24 e Å^−3^

### 

Data collection: *APEX2* (Bruker, 2004 ▶); cell refinement: *SAINT* (Bruker, 2004 ▶); data reduction: *SAINT*; program(s) used to solve structure: *SHELXS97* (Sheldrick, 2008 ▶); program(s) used to refine structure: *SHELXL97* (Sheldrick, 2008 ▶); molecular graphics: *SHELXTL* (Sheldrick, 2008 ▶); software used to prepare material for publication: *SHELXTL*.

## Supplementary Material

Crystal structure: contains datablocks I, global. DOI: 10.1107/S1600536810044478/rz2510sup1.cif

Structure factors: contains datablocks I. DOI: 10.1107/S1600536810044478/rz2510Isup2.hkl

Additional supplementary materials:  crystallographic information; 3D view; checkCIF report

## Figures and Tables

**Table 1:** Hydrogen-bond geometry (Å, °) *Cg*1 and *Cg*2 are the centroids of the C1–C6 and N1/C8–C12 rings, respectively.

*D*—H⋯*A*	*D*—H	H⋯*A*	*D*⋯*A*	*D*—H⋯*A*
N2—H2⋯N1^i^	0.86	2.24	3.0518 (19)	157
C12—H12⋯*Cg*1^i^	0.93	2.72	3.536 (2)	147
C4—H4⋯*Cg*2^ii^	0.93	3.14	3.804 (2)	130

Symmetry codes: (i) 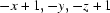; (ii) 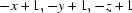.

**Fig. 1.**
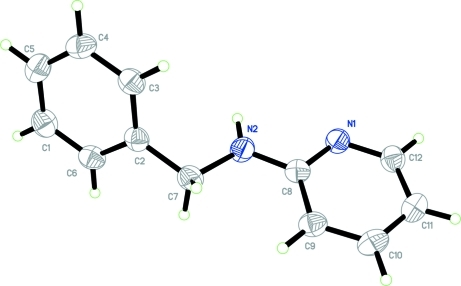
The molecular structure of the title compound showing the atom numbering scheme and 50% probability displacement ellipsoids.

**Fig. 2.**
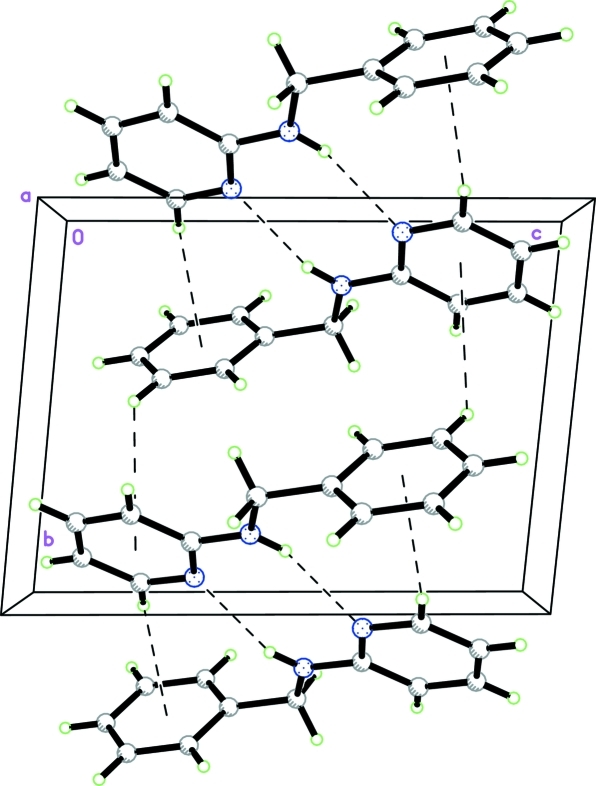
View of the three-dimensional structure of the title compound. Hydrogen bonds and C—H···π interactions are shown as dased lines.

## supplementary crystallographic information

### Comment

The design and construction of metal-organic frameworks (MOFs) is of great research interest due to their intriguing topologies and potential applications as functional materials (Benelli & Gatteschi, 2002). The 2-benzylaminopyridine ligand possessing two nitrogen donors to coordinate to metal ions, provides unique opportunities for the construction of various coordination networks. Recently, some complexes based on this ligand have been reported (Davies *et al.*, 2001; Wan *et al.*, 2004; Zhou & Richeson, 1995). When reacted with Ce(NO_3_)_3_ under hydrothermal condition, we isolated single crystals of the 2-benzylaminopyridine ligand, whose structure is reported herein.

The structure of the title compound is depicted in Fig. 1. The benzyl and the pyridyl rings are not coplanar and form a dihedral angle of 67.2 (1)°. The C—C and C—N bond lengths show normal values (Allen *et al.*, 1987). Intermolecular N—H···N hydrogen bonds (graph set motif *R*^2^_2_(8); Bernstein *et al.*, 1995) involving a centrosymmetrically related pair of molecules gives rise to a dimer, which is also stabilized by C—H···π stacking interactions (Table 1). C—H···π stacking interactions between neighbouring dimers further extend the structure to form a three-dimensional supramolecular network (Fig. 2).

### Experimental

A mixture of Ce(NO_3_)_3_.6H_2_O (0.163 g, 0.5 mmol), 2-benzylaminopyridine (0.092 g, 0.5 mmol), and H_2_O (10 mL) was sealed in a 15 mL Teflon-lined reactor, which was heated in an oven to 423 K for 24 h and then cooled to room temperature at a rate of 5 Kh^-1^. Colourless crystals were obtained in a yield of 58% based on 2-benzylaminopyridine.

### Refinement

All H atoms were fixed geometrically and treated as riding with C—H = 0.93-0.97 Å, N—H = 0.86 Å, and with U_iso_(H) = 1.2U_eq_(C, N).

### Crystal data

**Table:**

C_12_H_12_N_2_	*Z* = 2
*M**_r_* = 184.24	*F*(000) = 196
Triclinic, *P*1	*D*_x_ = 1.234 Mg m^−^^3^
Hall symbol: -P 1	Mo *K*α radiation, λ = 0.71073 Å
*a* = 5.9014 (16) Å	Cell parameters from 3415 reflections
*b* = 8.025 (2) Å	θ = 1.2–28.0°
*c* = 10.561 (3) Å	µ = 0.07 mm^−^^1^
α = 95.471 (4)°	*T* = 296 K
β = 91.244 (4)°	Block, colourless
γ = 94.779 (3)°	0.23 × 0.20 × 0.19 mm
*V* = 495.9 (2) Å^3^	

### Data collection

**Table:**

Bruker APEXII area-detector diffractometer	1387 reflections with *I* > 2σ(*I*)
Radiation source: fine-focus sealed tube	*R*_int_ = 0.012
graphite	θ_max_ = 25.3°, θ_min_ = 1.9°
φ and ω scans	*h* = −6→7
2551 measured reflections	*k* = −9→9
1762 independent reflections	*l* = −9→12

### Refinement

**Table:**

Refinement on *F*^2^	Primary atom site location: structure-invariant direct methods
Least-squares matrix: full	Secondary atom site location: difference Fourier map
*R*[*F*^2^ > 2σ(*F*^2^)] = 0.042	Hydrogen site location: inferred from neighbouring sites
*wR*(*F*^2^) = 0.115	H-atom parameters constrained
*S* = 1.07	*w* = 1/[σ^2^(*F*_o_^2^) + (0.0576*P*)^2^ + 0.0854*P*] where *P* = (*F*_o_^2^ + 2*F*_c_^2^)/3
1762 reflections	(Δ/σ)_max_ < 0.001
127 parameters	Δρ_max_ = 0.23 e Å^−^^3^
0 restraints	Δρ_min_ = −0.24 e Å^−^^3^

### Special details

**Table:**

Geometry. All esds (except the esd in the dihedral angle between two l.s. planes) are estimated using the full covariance matrix. The cell esds are taken into account individually in the estimation of esds in distances, angles and torsion angles; correlations between esds in cell parameters are only used when they are defined by crystal symmetry. An approximate (isotropic) treatment of cell esds is used for estimating esds involving l.s. planes.
Refinement. Refinement of F^2^ against ALL reflections. The weighted R-factor wR and goodness of fit S are based on F^2^, conventional R-factors R are based on F, with F set to zero for negative F^2^. The threshold expression of F^2^ > 2sigma(F^2^) is used only for calculating R-factors(gt) etc. and is not relevant to the choice of reflections for refinement. R-factors based on F^2^ are statistically about twice as large as those based on F, and R- factors based on ALL data will be even larger.

### Fractional atomic coordinates and isotropic or equivalent isotropic displacement parameters (Å^2^)

**Table:**

	*x*	*y*	*z*	*U*_iso_*/*U*_eq_	
C1	−0.1362 (3)	0.24905 (19)	0.36541 (15)	0.0350 (4)	
H1	−0.2427	0.1909	0.4122	0.042*	
C2	0.0725 (3)	0.30933 (18)	0.42172 (14)	0.0308 (4)	
C3	0.2280 (3)	0.39773 (19)	0.34991 (15)	0.0357 (4)	
H3	0.3689	0.4396	0.3860	0.043*	
C4	0.1748 (3)	0.4236 (2)	0.22596 (16)	0.0397 (4)	
H4	0.2798	0.4832	0.1793	0.048*	
C5	−0.0331 (3)	0.3616 (2)	0.17061 (16)	0.0408 (4)	
H5	−0.0680	0.3788	0.0868	0.049*	
C6	−0.1889 (3)	0.2740 (2)	0.24050 (16)	0.0403 (4)	
H6	−0.3292	0.2317	0.2038	0.048*	
C7	0.1283 (3)	0.2820 (2)	0.55703 (15)	0.0359 (4)	
H7A	0.1617	0.3897	0.6065	0.043*	
H7B	−0.0022	0.2242	0.5932	0.043*	
C8	0.4265 (3)	0.15939 (18)	0.67594 (14)	0.0301 (4)	
C9	0.3600 (3)	0.23242 (19)	0.79450 (14)	0.0353 (4)	
H9	0.2360	0.2968	0.7998	0.042*	
C10	0.4814 (3)	0.2067 (2)	0.90146 (15)	0.0412 (4)	
H10	0.4406	0.2545	0.9805	0.049*	
C11	0.6648 (3)	0.1099 (2)	0.89304 (15)	0.0397 (4)	
H11	0.7507	0.0930	0.9649	0.048*	
C12	0.7150 (3)	0.03973 (19)	0.77449 (15)	0.0359 (4)	
H12	0.8367	−0.0269	0.7683	0.043*	
N1	0.6010 (2)	0.06064 (16)	0.66736 (12)	0.0334 (3)	
N2	0.3221 (2)	0.18353 (16)	0.56430 (12)	0.0355 (3)	
H2	0.3734	0.1384	0.4947	0.043*	

### Atomic displacement parameters (Å^2^)

**Table:**

	*U*^11^	*U*^22^	*U*^33^	*U*^12^	*U*^13^	*U*^23^
C1	0.0330 (9)	0.0302 (8)	0.0414 (9)	0.0009 (6)	0.0061 (7)	0.0020 (7)
C2	0.0328 (8)	0.0249 (7)	0.0354 (8)	0.0081 (6)	0.0040 (6)	0.0010 (6)
C3	0.0314 (9)	0.0348 (9)	0.0405 (9)	0.0005 (7)	0.0013 (7)	0.0026 (7)
C4	0.0443 (10)	0.0352 (9)	0.0403 (9)	0.0000 (7)	0.0100 (7)	0.0075 (7)
C5	0.0503 (11)	0.0386 (9)	0.0340 (9)	0.0075 (8)	−0.0017 (7)	0.0041 (7)
C6	0.0358 (9)	0.0403 (9)	0.0431 (10)	0.0021 (7)	−0.0041 (7)	−0.0020 (7)
C7	0.0355 (9)	0.0356 (8)	0.0378 (9)	0.0096 (7)	0.0050 (7)	0.0037 (7)
C8	0.0329 (9)	0.0249 (7)	0.0326 (8)	0.0010 (6)	0.0036 (6)	0.0033 (6)
C9	0.0410 (10)	0.0312 (8)	0.0343 (9)	0.0064 (7)	0.0064 (7)	0.0015 (6)
C10	0.0544 (11)	0.0388 (9)	0.0301 (9)	0.0045 (8)	0.0067 (7)	−0.0003 (7)
C11	0.0481 (10)	0.0382 (9)	0.0323 (9)	0.0022 (8)	−0.0039 (7)	0.0043 (7)
C12	0.0355 (9)	0.0336 (8)	0.0389 (9)	0.0032 (7)	−0.0014 (7)	0.0055 (7)
N1	0.0357 (8)	0.0328 (7)	0.0322 (7)	0.0068 (6)	0.0009 (6)	0.0022 (5)
N2	0.0416 (8)	0.0377 (7)	0.0286 (7)	0.0147 (6)	0.0023 (6)	0.0012 (6)

### Geometric parameters (Å, °)

**Table:**

C1—C2	1.385 (2)	C7—H7B	0.9700
C1—C6	1.386 (2)	C8—N1	1.3502 (19)
C1—H1	0.9300	C8—N2	1.3559 (19)
C2—C3	1.395 (2)	C8—C9	1.409 (2)
C2—C7	1.500 (2)	C9—C10	1.366 (2)
C3—C4	1.378 (2)	C9—H9	0.9300
C3—H3	0.9300	C10—C11	1.383 (2)
C4—C5	1.381 (2)	C10—H10	0.9300
C4—H4	0.9300	C11—C12	1.372 (2)
C5—C6	1.380 (2)	C11—H11	0.9300
C5—H5	0.9300	C12—N1	1.3353 (19)
C6—H6	0.9300	C12—H12	0.9300
C7—N2	1.448 (2)	N2—H2	0.8600
C7—H7A	0.9700		
			
C2—C1—C6	121.05 (15)	C2—C7—H7B	109.5
C2—C1—H1	119.5	H7A—C7—H7B	108.0
C6—C1—H1	119.5	N1—C8—N2	115.92 (13)
C1—C2—C3	118.30 (14)	N1—C8—C9	121.27 (14)
C1—C2—C7	120.76 (14)	N2—C8—C9	122.81 (14)
C3—C2—C7	120.94 (14)	C10—C9—C8	118.62 (15)
C4—C3—C2	120.63 (15)	C10—C9—H9	120.7
C4—C3—H3	119.7	C8—C9—H9	120.7
C2—C3—H3	119.7	C9—C10—C11	120.44 (15)
C3—C4—C5	120.50 (16)	C9—C10—H10	119.8
C3—C4—H4	119.8	C11—C10—H10	119.8
C5—C4—H4	119.8	C12—C11—C10	117.34 (15)
C6—C5—C4	119.57 (15)	C12—C11—H11	121.3
C6—C5—H5	120.2	C10—C11—H11	121.3
C4—C5—H5	120.2	N1—C12—C11	124.42 (15)
C5—C6—C1	119.96 (15)	N1—C12—H12	117.8
C5—C6—H6	120.0	C11—C12—H12	117.8
C1—C6—H6	120.0	C12—N1—C8	117.85 (13)
N2—C7—C2	110.93 (13)	C8—N2—C7	122.91 (13)
N2—C7—H7A	109.5	C8—N2—H2	118.5
C2—C7—H7A	109.5	C7—N2—H2	118.5
N2—C7—H7B	109.5		

### Hydrogen-bond geometry (Å, °)

**Table:**

Cg1 and Cg2 are the centroids of the C1–C6 and N1/C8–C12 rings, respectively.

**Table:**

*D*—H···*A*	*D*—H	H···*A*	*D*···*A*	*D*—H···*A*
N2—H2···N1^i^	0.86	2.24	3.0518 (19)	157
C12—H12···Cg1^i^	0.93	2.72	3.536 (2)	147
C4—H4···Cg2^ii^	0.93	3.14	3.804 (2)	130

Symmetry codes: (i) −*x*+1, −*y*, −*z*+1; (ii) −*x*+1, −*y*+1, −*z*+1.

## References

[bb1] Allen, F. H., Kennard, O., Watson, D. G., Brammer, L., Orpen, A. G. & Taylor, R. (1987). *J. Chem. Soc. Perkin Trans. 2*, pp. S1–19.

[bb2] Benelli, C. & Gatteschi, D. (2002). *Chem. Rev.* **102**, 2369–2388. 10.1021/cr010303r 12059272

[bb3] Bernstein, J., Davis, R. E., Shimoni, L. & Chang, N.-L. (1995). *Angew. Chem. Int. Ed.* **34**, 1555–1573.

[bb4] Bruker (2004). *APEX2* and *SMART* Bruker AXS Inc, Madison, Wisconsin, USA.

[bb5] Davies, R. P., Linton, D. J., Schooler, P., Snaith, R. & Wheatley, A. E. H. (2001). *Chem. Eur. J.* **7**, 3696–3704. 10.1002/1521-3765(20010903)7:17<3696::aid-chem3696>3.0.co;2-o 11575770

[bb6] Sheldrick, G. M. (2008). *Acta Cryst.* A**64**, 112–122. 10.1107/S0108767307043930 18156677

[bb7] Wan, C.-Q., Li, Q.-S., Song, H.-B., Xu, F.-B. & Zhang, Z.-Z. (2004). *Acta Cryst.* E**60**, m1973–m1975.

[bb9] Wang, G. G. & Zhao, H. (2010). *Acta Cryst.* E**66**, o3077. 10.1107/S1600536810044351 PMC3011657 21589386

[bb8] Zhou, Y. & Richeson, D. S. (1995). *Organometallics*, **14**, 3558–3561.

